# Correction to Therapeutic effects of mesoderm introduction of compound glycyrrhizin injection on the treatment of rosacea

**DOI:** 10.1111/srt.13525

**Published:** 2023-11-16

Li S, Zhao X, Chen Y, Liu J. Therapeutic effects of mesoderm introduction of compound glycyrrhizin injection on the treatment of rosacea. *Skin Res Technol*. 2023;29:e13328. https://doi.org/10.1111/srt.13328

In the “Funding Information” section, the text “The authors received no specific funding for this work.” was incorrect. This should have read: “This study was supported by Xintai Key Research Project (2021ZC132).”

We apologize for this error.

